# AGE DISPARITIES IN EXPOSURE TO HEAT WAVES, EXTREME WEATHER, AND WILDFIRES IN A NATIONAL UNITED STATES SAMPLE

**DOI:** 10.1093/geroni/igae098.0759

**Published:** 2024-12-31

**Authors:** Roger Wong

**Affiliations:** State University of New York Upstate Medical University, New York, United States

## Abstract

Severe climate events have become increasingly frequent. Extensive research has shown that older adults are more adversely impacted by these climate events, often due to more prevalent health conditions contributing to functional impairment. There is, however, no existing research that has examined whether age disparities may exist across exposures to different types of climate events, especially with a national sample. To address this gap, the 2022 American Trends Panel data were analyzed to examine age group differences in exposure to heat waves, extreme weather (flood or intense storm), and wildfires in a nationally representative U.S. sample of 10,282 adults aged 18 and older. In a multiple logistic regression model, adults aged 65+ had 42% significantly lower odds of exposure to heat waves compared to adults aged 18-29 (adjusted odds ratio [aOR]=0.58, 95% CI=0.45-0.76, p<.001), after applying survey sampling weights and adjusting for a variety of sociodemographic covariates. For extreme weather exposure, adults aged 65+ had 44% significantly lower odds compared to adults aged 18-29 (aOR=0.56, 95% CI=0.43-0.72, p<.001). The odds of wildfire exposure was 45% significantly lower among adults aged 65+ compared to adults aged 18-29 (aOR=0.55, 95% CI=0.38-0.79, p<.01). Across all age groups, our results indicate older adults are the least likely to be exposed to climate events related to heat, extreme weather, and wildfire despite the higher strain placed on this populations. Future research is warranted to understand factors contributing to lower climate event exposures, and degree of burden when these different climate events occur among older populations.

